# Telehealth For Individuals with Parkinson's Disease During Covid-19 In Brazil: A Prospective Case Series

**DOI:** 10.5195/ijt.2022.6471

**Published:** 2022-12-13

**Authors:** Natália Mariano Barboza, Hayslenne Andressa Gonçalves de Oliveira Araújo, Marcelle Brandão Terra, Maria Eduarda Brandão Bueno, Rogério José de Souza, Andressa Letícia Miri, Suhaila Mahmoud Smaili

**Affiliations:** 1 PT, MSc, Neurofunctional Physical Therapy Research Group (GPFIN), Graduate program in Rehabilitation Sciences – State University of Londrina, Paraná, Brazil; 2 PT, PhD, Department of Physiotherapy, Neurofunctional Physical Therapy Research Group (GPFIN), Master's and Doctoral degree program in Rehabilitation Sciences – State University of Londrina, Paraná, Brazil

**Keywords:** Coronavirus infections, Parkinson's disease, Physiotherapy, Telerehabilitation

## Abstract

**Objective:**

To implement a telerehabilitation prevention, treatment, and follow-up physical therapy protocol for monitoring individuals with Parkinson's disease (PD) and to verify its effectiveness in minimizing the deleterious effects of the COVID-19 pandemic.

**Design:**

Prospective case series, involving 40 participants with mild to moderate PD recruited from a specialized neurorehabilitation group. The study was divided into four parts: (1) Phone calls to assess the feasibility of participating in remote physical therapy. (2) Social media training. (3) Baseline and post-intervention assessment for functional lower extremity strength, fear of falling, quality of life, depression, anxiety, activities of daily living, verbal fluency. (4) Intervention protocol consisting of 20 remote weekly physical therapy sessions, graphic material for physical and cognitive training, social activities, and education.

**Conclusion:**

The telerehabilitation protocol was viable and effective for patients with PD as an alternative to in-person treatment during the COVID-19 pandemic.

Due to the novel coronavirus (COVID-19) pandemic, the World Health Organization (WHO) declared a global emergency. To prevent the spread of COVID-19 due to its high transmissibility, WHO recommended frequent hand washing, use of personal protective equipment such as masks, avoiding contact with symptomatic individuals, and social isolation (Sohrabi et al., 2020).

Social isolation and even “lockdown” were strategies implemented to contain the pandemic. These approaches confine individuals to their homes for long periods, which limits social contact with family and friends. Elderly people, particularly those who are fragile or are considered to be high risk in relation to COVID-19, need more severe isolation, resulting in an increase in sedentary behavior, deconditioning, balance disorders, increased risk of falls, and worsening or emergence of new mental and social problems such as loneliness, negative economic impact and worse quality of life (de Biase et al., 2020).

Regarding the neurological consequences caused by the new coronavirus and its possible impact on patients with neurodegenerative conditions, studies indicate that patients with Parkinson's disease (PD), especially those who are older and those with longer duration of diagnosis may represent a population more vulnerable to contagion (van Wamelen et al., 2020). In particular, this population may experience more severe symptoms of COVID-19 due to a decrease in range of movement and trunk mobility, respiratory muscle rigidity, impaired phonation, respiratory and swallowing incoordination, and impaired cough reflex in conjunction with preexisting dyspnea (Ellul et al., 2020; van Wamelen et al., 2020). In addition, possible indirect effects resulting from restrictive measures and lockdown may arise, such as increased stress, depression, self-isolation and anxiety, as well as the consequences of prolonged immobility (e.g., altered postural balance, weakness, and muscle shortening), falls and fear of falling, gait impairment, deprivation of cognitive stimulation, dementia and loss of independence and autonomy (Antonini et al., 2020).

Patients with PD need routine hospital and clinic visits to adjust medication and participate in rehabilitation activities by movement disorders specialists, however, it was recommended to avoid presential visits to clinics during this period. Fortunately, telehealth was identified as a viable option for patients with PD (Cubo et al., 2020; Papa et al., 2020) and its implementation increased rapidly. Telehealth can be used for routine follow-up, urgent visits, new consultations, research visits, psychotherapy, genetic counseling, social services, rehabilitation, and education (Cubo et al., 2020). Because telehealth involves remote delivery of health care services using telecommunications technology, access to care is increased, allowing for continuation of appropriate patient care.

Due to the COVID-19 pandemic, rapid changes were necessary in the field of rehabilitation that required new models of care via telerehabilitation (Haines & Berney, 2020). In this sense, the Brazilian Federal Council of Physiotherapy has authorized and regulated telehealth for physical therapists (*Conselho Federal de Fisioterapia e Terapia Ocupacional (COFFITO)*, n.d.). Telephone consultations, video visits or other forms of virtual care were, more than ever, welcomed. These required internet connection, availability of technology, and technological knowledge to use the devices (i.e., digital inclusion). Planning, training, and well-established protocols on the part of professionals were essential (Bettger et al., 2020).

To minimize the deleterious effects described above, we proposed new strategies to monitor individuals with PD who had previously been regularly treated in person. As such, this study had two aims. First, we proposed an unprecedented telehealth protocol for individuals with PD. Second, we verified the protocol's effectiveness on quality of life, depression, anxiety, fear of falling, activities of daily living, motor functionality, and cognition. We hypothesized that the telerehabilitation intervention protocol would increase or maintain the functional outcomes in the patients with PD.

## Methods

### Participants

The sample was characterized by a convenience sample, composed by individuals with PD, belonging to the Physical Therapy Ambulatory Specialized in PD at the State University of Londrina (UEL), which is associated with the Neurofunctional Physical Therapy Research Group (GPFIN) that has been developing its activities since the year 2010, in Londrina – Paraná – Brazil.

The following inclusion criteria were applied: diagnosis of idiopathic PD confirmed by a movement disorders specialist in accordance with Parkinson's disease Society Brain Bank diagnostic criteria (Hughes et al., 1992); age over 50 years; disease staging between 1.5 and 3 according to the modified Hoehn and Yahr scale (Hoehn & Yahr, 1967); undergoing regular and stable pharmacological treatment; Mini-Mental State Examination score ≥ 24 (Folstein & Folstein, 1975); no other associated neurological, musculoskeletal and/or cardiorespiratory diseases, and the ability to walk independently. All the inclusion criteria mentioned above were requirements for participation in the GPFIN group. As the participants were previously part of that group, they already met these criteria. In addition, to be included in this protocol we required: having a smartphone or computer and access to internet; knowledge about or available assistance on how to make video calls; and the ability to participate and provide written informed consent.

The exclusion criteria consisted of a diagnosis of atypical PD; neuropsychiatric comorbidities; inability to walk 10 meters; presence of severe dyskinesia that prevents the participant from sitting in a chair; not understanding any of the training protocol stages and experiencing severe pain and/or discomfort that precludes performing the proposed activities.

### Design

A prospective case series was conducted via telehealth. The project was approved by the Institutional Research Ethics Committee (3.353.856) and addressed the Declaration of Helsinki.

The study was divided into four parts. The first part consisted of phone calls to know how individuals were dealing with the COVID-19 pandemic and to consider the possibility of carrying out the study by means of online physical therapy. We asked the following standardized questions through telephone calls: (a) How is the quarantine period going? (b) What, if any, major events have occurred during this period? (i.e., falls, changes in medication, surgery, etc.); (c) How is the practice of physical activity progressing during quarantine? (d) Can they participate in an online approach? (e) Is internet access available? and (f) Can they use technology for telehealth services?

The second part consisted of social media use training through informative videos and telephone calls to assist patients in handling the equipment (computer or cell phone) as well as applications such as WhatsApp and Google Meet. This step was significatively important considering that access to technology is not always widely available for all people in Brazil, especially for the older population.

The third part consisted of assessment procedures via telephone contact. The evaluation order was randomized (www.sealedenvelope.com) by an external person. The evaluator assessed the same patient pre- and post-intervention. More details about the assessment procedures are provided below. At the end of the intervention, we also asked patients about their behavior during the isolation period and how they felt regarding their PD related symptoms. Patients were asked to report their habits (such as performance of exercises, level of contact with friends and family, health support) as well as their PD related symptoms (for instance worsening tremor, rigidity, bradykinesia, gait, balance, etc.) in a yes/no questionnaire developed by our group.

The fourth part consisted of online physiotherapy intervention procedures carried out with the objectives of prevention, rehabilitation, and health promotion. The telehealth protocol was comprised of social activities, online physiotherapy sessions, printed materials with physical and cognitive activities, and educational lectures.

### Screening

Participants were screened via an initial telephone call before being assessed for the stated outcomes. A structured evaluation was carried out that included sociodemographic data; time since diagnosis; disease severity according to the modified Hoehn and Yahr Clinical Staging Scale; and levodopa equivalent daily dose (LEDD).

Screening also assessed the intention to participate in the study, availability of internet access, and familiarity with the use of cell phone and computer technologies.

### Assessment Procedures and Outcomes

The third part of the protocol consisted of the patient's evaluation. The instruments used and the outcomes assessed were as follows: (1) Five Times Sit-to-Stand (5TSTS) to quantify functional lower extremity strength and identify movement strategies used to complete transitional movements (Duncan et al., 2011); (2) Falls Efficacy Scale (FES-I) to assess fear of falling (Camargos et al., 2010); (3) Parkinson's Disease Questionnaire (PDQ-39) to assess quality of life (Souza et al., 2007); (4) Hospital Anxiety Depression Scale (HADS) to measure symptoms of depression and anxiety (Faro, 2015); (5) MSD – Unified Parkinson's Disease Rating Scale part two (MDS-UPDRS II) to assess activities of daily life (ADL) (Goetz et al., 2008); (6) Verbal Fluency (VF) Test to assess semantic and phonemic fluency in 1 minute (Rodrigues et al., 2008). The choice of the tests was due to the possibility of performing them by telephone call.

Assessments were carried out during the “on” stage of medication, always at the same time of day, and by the same evaluator. The order of test application was randomized (www.sealedenvelope.com) with the same order on pre- and post-evaluation moments.

### Telehealth intervention procedures

#### Social activities

The first and the last sessions of the protocol were a commemorative activity with all individuals participating simultaneously in an online meeting using the Google Meet application. The main objective of these two sessions was to improve social interactions between participants. The sessions consisted of games, music, interaction, and other social activities.

#### Online physiotherapy sessions

Patients attended 20 supervised physical therapy sessions, once a week, for 60 minutes. Subjects were divided into groups of three based on disease staging and disease subtype (akinetic-rigid, tremulant, or mixed) to make homogeneous groups for the physical therapy sessions. Each physical therapist cared for a group of three patients in the period of four weeks. After that, another physical therapist led the group. By the end of the protocol, five physical therapists passed through each group. The physical therapist who carried out the evaluations were not the same ones who carried out the interventions. All interventions happened online via WhatsApp video call or Google Meet.

The exercise sessions were designed according to a specific objective each week, namely: exercises for upper limbs and upper trunk (sessions 1, 5, 9, 13, 17); exercises for lower limbs and lower trunk (sessions 2, 6, 10, 14, 18); dual cognitive and motor tasks (sessions 3, 7, 11, 15, 19); and balance and gait exercises (sessions 4, 8, 12, 16, 20).

Each objective was repeated after four weeks. The exercises had an increased degree of complexity (from 1 to 5, with 1 being the easiest and 5 the most difficult) during the sessions. The sessions were comprised of 50-minute exercises according to the aim of the week and 10 minutes of stretching. Patients were required to either perform 10 repetitions of each exercise or hold the exercise position for 30 seconds. Afterwards, they were allowed to rest for 30 seconds. This study design is shown in the Appendix. All exercises performed at these supervised online sessions were described beforehand in a graphic material (printed) that was delivered to the patients with instructions for hygiene and protection care for physical contact as suggested by the Brazilian Ministry of Health. In addition, all exercises were filmed and sent as videos to the patients.

#### Printed material with physical and cognitive activities

Graphic material in the form of a booklet was developed containing ten exercises to be performed daily. These were similar to those performed in the online supervised sessions, with the same therapeutic objective and the same level of complexity as the aforementioned weeks. We recommended that patients repeat the exercises of the week, daily. Furthermore, a list of daily stretching exercises was provided as well.

In addition to motor exercises, three cognitive paper-pencil activities per week were provided. The activities consisted of completing mazes, performing calculations, identifying images in different backgrounds, memory activities, and language, among others. Moreover, access to weekly cognitive training was provided through a link to internet programs. The objectives of the paper-pencil and online cognitive activities was to train all the cognitive domains, including memory, executive function, language, attention, etc.

In summary, in addition to weekly online supervised physical therapy, individuals were able to perform motor and cognitive exercises through graphic materials delivered to their homes at the beginning of the study. The daily performance (or not) of the exercises described above during the week (except on the day of the remote physical therapy) was recorded on a sheet developed to monitor the attendance of participants in these activities.

#### Educational lectures

A monthly online lecture via Google Meet was held with all patients, physical therapists, and the project coordinator with the aims of providing adequate information in relation to COVID-19, helping participants to manage and deal with specific symptoms of PD due to social isolation, and providing an open space for participants to give their feedback for potential adjustments to these activities, if necessary. The topics that were discussed included: information about COVID-19; prevention of falls; how to deal with emotions during the pandemic; physical exercise in PD; and the family's role in PD rehabilitation.

### Participant loss and adherence

Patients were considered lost to follow up when there were: more than 20 percent of absences during the training sessions; changes in medication throughout the intervention; missing post-intervention evaluations; or illness that prevented continuity in the study. Adherence strategies, such as telephone contact with participants, were used to remind them of the evaluation and intervention sessions. The hours offered were flexible, and attempts were made to prevent or resolve possible problems that could interfere with participation and continuity in the intervention.

### Safety

The intervention presented minimal risks to participants, although there are some inherent risks associated with age, such as changes in blood pressure, signs of fatigue, tiredness, or muscle or joint pain. To control adverse effects, patients were asked to identify the sensations and any discomfort experienced during the sessions. To minimize and manage these risks, the physical therapists monitored the signals. This instruction was standardized to avoid fatigue and keep the protocol consistent. As prior physical therapy patients they were already familiar with the proposed exercises, which minimized the risks of intervention. Patients were instructed to always have someone by their side during physical therapy sessions to ensure their safety. No adverse events were reported during the intervention.

## Data analysis

Results are presented as mean (standard deviation) or median (interquartile range) according to a normality distribution, analyzed by Shapiro-Wilk test, as well as Levene's variance homogeneity test. The pre- and post-intervention moments were compared using the t-test or Wilcoxon test, according to normality distribution, and analyzed by Shapiro-Wilk test. The effect size (d Cohen) was calculated and characterized as: small (*d*=0.0–0.20), medium (*d*=0.30–0.50), or large (*d*=0.50–0.80). The Microsoft Excel 2010 (Microsoft, EUA) and Statistical Package for the Social Sciences (SPSS) version 27.0 (IBM, EUA) were used in data tabulation and data analysis respectively, and a 5% alpha (p<0.05) was established. All statistics were performed according to intention-to-treat analyses.

## Results

In total, 47 participants were considered potentially eligible and were examined for eligibility. Seven were excluded due to: not wanting to participate remotely (n=3) or not having access to internet connection (n=4). As a result, 40 subjects were included and initiated the protocol. During the 20 weeks of intervention, there was attrition of 6 participants due to: moving to a place without internet connection (n=1); having more than 20 percent absences of physical therapy sessions (n=3); deciding not to participate anymore (n=1); and undergoing an abdominal surgery (n=1). Thereby, 34 individuals finished the protocol. Baseline characteristics of the participants are shown in Table 1.

**Table 1:** Participant Characteristics

	Participants (n=40)	
	Mean	SD
Gender (F/M)	14/26	
Age	69.18	9.26
Disease duration (years)	7.20	4.97
LEDD	835.30	494.68
Years of schooling	10.64	5.23

*Note.* F: female/M: male/LEDD: Levodopa equivalent daily dose

The dropout rate was 6 individuals (15%). Considering that 34 individuals performed the entire protocol (i.e., 20 sessions), we provided 680 sessions in total. Considering also that the one-hour weekly session should be multiplied by 6 times a week (patients were instructed to perform the same exercises at home), the protocol offered more than 4080 opportunities to exercise to the participants. Moreover, 60 options of cognitive exercises were provided for each patient and 5 sessions of group health education.

Considering the baseline and after protocol intervention, differences between the pre and post intervention moments in the 5TSTS (p=0.010/*d*=0.33) and in the VF Test (p=0.027/*d*=0.39) were found. During the follow-up at 20 weeks, patients improved their lower extremity functional strength and cognition. There was no statistically significant difference for the outcomes of fear of falling, quality of life, anxiety, depression, and PD symptoms in ADL (Table 2).

**Table 2:** Outcomes Measures at Baseline and Changes after Intervention Protocol

	Pre intervention	Post intervention	p value	ES
5TSTS	15.11 (4.20)	13.72 (3.75)	0.010^*^	0.33
FES-I	32.00 [22.00 – 40.00]	32.00 [24.00 – 41.00]	0.702	0.06
PDQ-39	30.51 (12.14)	30.31 (14.23)	0.761	0.01
Mobility	35.70 (22.01)	35.32 (24.48)	0.872	0.02
ADL	33.22 (22.41)	32.90 (20.76)	0.914	0.01
Emotional well-being	28.95 (18.41)	29.49 (18.37)	0.627	0.02
Stigma	12.50 [0.00 – 25.00]	6.25 [0.00 – 18.75]	0.652	0.07
Social support	16.67 [8.33 – 33.33]	25.00 [0.00 – 41–67]	0.133	0.23
Cognition	34.29 (18.13)	33.81 (19.80)	0.678	0.02
Communication	16.67 [8.33 – 33.33]	16.67 [8.33 – 33.33]	0.755	0.04
Bodily discomfort	44.44 (23.60)	42.31 (26.45)	0.799	0.09
HADS	11.05 (5.51)	11.15 (5.98)	0.633	0.01
HADS - A	5.44 (2.99)	5.46 (3.23)	0.695	0.00
HADS - D	5.62 (3.24)	5.69 (3.48)	0.796	0.02
MDS UPDRS II	14.31 (7.59)	14.18 (7.63)	0.940	0.01
VF	15.64 (4.07)	17.23 (4.91)	0.027^*^	0.39

*Note*. ES: Effect size 5TSTS: Five Times Sit to Stand/FES-I: Falls Efficacy Scale – International/PDQ-39: Parkinson's Disease Questionnaire – PDQ-39/ADL: Activities of daily living/HADS: Hospital Anxiety and Depression Scale/HADS – A: Hospital Anxiety and Depression Scale – Anxiety/HADS – D: Hospital Anxiety and Depression Scale – Depression/MDS – UPDRS II: Movement Disorder Society – Unified Parkinson's Disease Rating Scale II/VF: Verbal Fluency

Data resulted from the questionnaire developed by the GPFIN group showed the participants' routine and the events that occurred during the monitoring pandemic period (Table 3), as well as the patient's perception in relation to the signals and symptoms of PD (Table 4). We observe that patients prioritized going to medical appointments and meeting with their families. During the follow up period, no patients had been infected by COVID-19 and about 50% managed to maintain physical activity. When asked about PD related symptoms, the participants mentioned that they felt worse during this period regarding bradykinesia, gait, and balance mainly. These data helped the professionals involved in the telerehabilitation protocol manage the conditions presented by the participants.

**Table 3:** Isolation Period Activities

	Yes	No
Practice of physical activity	55.17%	44.83%
Meeting with family	72.41%	27.59%
Meeting with friends	51.72%	48.28%
Participation in religious activities	34.48%	65.52%
Participation in community activities	6.90%	93.10%
Going to medical appointments	82.76%	17.24%
Receiving health support	31.03%	68.97%
Positive test for COVID-19	0%	100%
Necessity of hospitalization	6.90%	93.10%

**Table 4:** Worsening of Symptoms Related to PD

	Yes	No
Shaking	20.69%	79.31%
Balance	51.72%	48.28%
Rigidity	41.38%	58.62%
Bradykinesia	55.17%	44.83%
Walking	51.72%	48.28%
Falls	48.28%	51.72%
Fatigue	44.83%	55.17%
Sleep	31.03%	68.97%
Memory	48.28%	51.72%
Depressive Symptoms	34.48%	65.52%
Pain	44.83%	55.17%

## Discussion

This is the first study in Brazil to develop and implement a telerehabilitation treatment protocol in a population with PD using a synchronous approach. This is important because of the following factors: (1) As PD is a chronic and progressive disease, a team of health care professionals was required to put this protocol into practice; (2) Digital inclusion, especially in an elderly population in Brazil, remains a huge challenge. However, this barrier has been broken despite the discrepant social, economic, and educational differences that exist; (3) The vast majority of telerehabilitation programs reported are asynchronous. We entered patients' homes weekly in a synchronous way and provided the support necessary to maintain the activities that had been performed in person before the advent of COVID-19 pandemic; (4) The report of our experiences can serve as a model for programs with a similar format (especially in developing countries); (5) Even at a distance, it was possible to measure (quantitatively) an individual's functionality using the 5TSTS test, fear of falling using FES-I; quality of life with PDQ-39; anxiety and depression with HADS; activities of daily living with MDS-UPDRS II; and verbal fluency through the VF test.

Our results showed improved functionality and verbal fluency after the intervention. Considering functionality, we can infer that an obvious explanation is because all the sessions involved trunk/lower extremity training and functional training such as postural changes, (including sit-to-stand training), due to its importance in individuals' independence. Therefore, this protocol was effective for our primary aim and can be implemented in clinical practice. However, the improvement in 5TSTS performance did not directly impact falls, quality of life, anxiety/depression and MDS-UPDRS scores, probably because they are multidimensional outcomes and many characteristics can determine them.

The other outcomes did not show improvements after the protocol. However, even though they were not the primary targets of the intervention, the non-worsening of these outcomes may be considered a satisfactory result, as we were dealing with a neurodegenerative and progressive disease in an unconventional pandemic period that may cause restricted mobility, activities, and social participation. Thus, this protocol, as the only alternative approach in this period, can be considered an innovative and effective form of treatment with satisfactory results.

As the SARS-CoV-2 virus continues to spread across the globe, it is still necessary to take measures to avoid infection and consequent deaths. Social distancing and lockdown are strategies used by some countries (Helmich & Bloem, 2020). Even after the initial threat of the pandemic, we will likely have a need for continued restrictions with our public and social lives for a time (i.e., months or even years), until vaccination (and strengthening vaccine) reach the world-wide population (Cubo, & Hassan, 2020).

In the field of neurorehabilitation, previous studies have shown beneficial effects of telerehabilitation. Ypinga et al. (2018) demonstrated that specialized physiotherapy delivered in a remote way to patients with PD through the program ParkinsonNET was associated with fewer hospital visits or admissions due to fractures or other orthopedic injuries, or pneumonia. It resulted in greater continuity of care, greater efficiency, lower costs, and reduced mortality. Isernia et al. (2020) reported the positive influence of a multidimensional rehabilitation approach performed at home for patients with PD on motor and non-motor functioning, in addition to improved quality of life and well-being, and lower societal costs. In contrast, the current study had a previous phase of in-clinic rehabilitation and assessments. The aforementioned studies were carried out in developed countries, which differs from ours in regard to availability of infrastructure (i.e., equipment and connectivity). Another different aspect is that in the current study, we observed patients in their natural environments since we carried out a synchronous program that we considered to be the ideal scenario.

One of the main barriers we faced in implementing the telerehabilitation protocol was the level of digital inclusion required by the elderly and neurologically pathologic population we were treating. We carried out detailed training to teach the patients how to handle this technology. This was a challenging process for a group that had been performing its activities for more than 10 years in-person, but an effort that was necessary to meet the challenges of the pandemic. Once they were familiar with it, we were surprised by how much it helped minimize the effects of solitude and isolation provoked by the disease. Patients could be closer and better connected to their families and friends than before.

Considering this, we hypothesized that a protocol based on telehealth including exercises, cognitive training, education, and social activities could be beneficial in terms of quality of life, anxiety, depression, lower extremity functionality, fear of falls reduction, daily living PD symptoms and cognition.

Possible explanations for our findings related to improvement/non-worsening of outcomes that were studied include: (1) the possibility of continuing treatment and remaining active, ensuring access to physical therapy; (2) the team support, which allowed patients to feel safe and to receive the necessary care in an online and synchronous way; (3) the feeling of belonging to a group and being in contact with the physical therapists and other patients each week; and, (4) an established network for support, since we were able to make quick decisions, determine the need for a medical appointment, and provide space for patients to speak about their PD and pandemic related hardships.

To our knowledge, in-person physical therapy sessions are more effective and allow better control than telehealth sessions. However, we have identified a good and even necessary option to not leave patients without treatment during the pandemic. Future studies should be designed to compare online and in-person rehabilitation. However, for the time being, the telerehabilitation protocol developed here serves as an effective tool with good cost-effective results.

Taking this into account, our telerehabilitation protocol's positive aspects include: the acquisition of digital inclusion in elderly people with PD; the significant adherence to an online rehabilitation approach; the improvement of functional and cognitive aspects; the maintenance of quality of life, performance on daily living activities, levels of depression, anxiety, fear of falling; the alternative possibility in the management of PD; and group support provided during a challenging period of the pandemic.

As limitations of this study, we note that the assessment procedure included non-gold standard instruments (that could be applied using telephone calls), lack of a control group, and difficulty in enrolling individuals without internet access and support to obtain that.

For future research, randomized controlled trials are needed to add more robust results to our findings, especially comparing in-person and remote treatment protocols. Furthermore, this study presents an opportunity to develop similar protocols that focus on falls, quality of life, anxiety/depression, and other motor outcomes using more robust measurement instruments (i.e., gold standard). As such, the protocol was effective for functional motor and cognitive outcomes and for maintaining the other outcomes assessed. It created the possibility for individuals to be followed by a specialized rehabilitation team even during COVID-19 pandemic.

## Appendix Intervention Description

**Table:**

Session	Objective	Level of difficulty	Physical therapist
Session 1	exercises for upper limbs and upper trunk	1	Physical therapist 1
1) Shoulder flexion and extension/2) Elbows flexion and extension/3) Exercise 1 and 2 combination/4) Shoulder horizontal abduction and adduction/5) Shoulder abduction and adduction/6) Exercise 4 and 5 combination/7) Trunk flexion and extension/8) Trunk rotation/9) Exercise 7 and 8 combination/10) Trunk rotation associated with shoulder flexion			
Session 2	exercises for lower limbs and lower trunk	1	Physical therapist 1
1) Anterior and posterior pelvic tilt/2) Lateral pelvic tilt/3) Hip flexion associated with opposite upper extremity extension/4) Hip abduction and adduction associated with opposite upper extremity movement/5) Knee extension associated with opposite upper extremity flexion 6) Ankle plantar flexion/7) Toes extension/8) Exercise 3 and 6 associated/9) Exercise 4 and 7 associated/10) Exercise 5 with crossing legs associated			
Session 3	dual cognitive and motor tasks	1	Physical therapist 1
1) Cervical flexion and extension + naming colors/2) Trunk rotation followed by side step + women names to the right and men names to the left/3) Trunk rotation followed by opposite leg side step + subtraction by threes/4) Trunk rotation followed by opposite knee extension + counting from two to the right and from 3 to the left/5) Rise to toes with shoulder flexion + naming animals/6) Shoulder and hip flexion + naming cities/7) Trunk anterior flexion associated to ankle plantar flexion + naming fruits/8) Sit to stand + naming countries/9) Knee and shoulder flexion + singing/10) Step forward followed by opposite side rotation trunk + subtraction by fours			
Session 4	balance and gait exercises	1	Physical therapist 1
1) Balance maintenance in Romberg position/2) Balance maintenance in Tandem position/3) Balance maintenance in one leg stance position/4) Anteroposterior limits of stability with reach exercise/5) Mediolateral limits of stability with reach exercise/6) Rise to toes alternating sides/7) Forward and backward leg oscillation/8) Lateral leg oscillation/9) Diagonal leg oscillation/10) Circumduction leg oscillation			
Session 5	exercises for upper limbs and upper trunk	2	Physical therapist 2
1) Coordination fingers and hand exercises involving snap fingers and clap hands in different positions/2) Coordination exercises using a stick moving away and bringing hands together/3) Exercise 2 crossing hands/4) Exercise 3 including supination and pronation/5) Trunk rotation associated with shoulder flexion/6) Trunk anterior inclination/7) Exercise 6 associated with trunk rotation/8) Exercise 6 associated with shoulder flexion/9) Trunk lateral flexion/10) Exercise 6, 8 and 9 associated.			
Session 6	exercises for lower limbs and lower trunk	2	Physical therapist 2
1) Hip flexion associated with opposite upper extremity extension in standing position/2) Hip extension associated with opposite upper extremity flexion in standing position/3) Hip abduction associated with opposite upper extremity movement in standing position/4) Squats/5) Knee extension associated with elbow flexion in standing position/6) Hip flexion associated with shoulder abduction/7) Coordination movements associating hip flexion, abduction and extension/8) Rise to toes/9) Exercise 4 with greater range of movement/10) Exercise 9 and 8 associated			
Session 7	dual cognitive and motor tasks	2	Physical therapist 2
1) Cervical rotation + naming kitchen objects to the right and bedroom objects to the left/2) Coordination exercise involving supination and pronation + singing/3) Coordination exercise combined with parts of the body + singing/4) Trunk rotation + counting from twos to the right and threes to the left/5) Shoulder abduction and adduction passing a ball from one hand to the other + people names beginning with letter M/6) Trunk flexion and extension associated with rotation + counting from fives to the right and from tens to the left/7) Sit to stand in Tandem position associated with trunk rotation + naming cars/8) Step forward associated with opposite upper extremity movement + naming colors to the right and fruits to the left/9) Side step associated with shoulder abduction + naming states to the right and countries to the left/10) Side steps associated with shoulder flexion passing a ball from one hand to the other + naming school objects			
Session 8	balance and gait exercises	2	Physical therapist 2
1) Shoulder abduction and adduction and flexion and extension passing a ball from one side to the other modifying center of gravidity/2) Small squats in Romberg position associated with shoulder flexion and extension modifying center of gravidity/3) Exercise 2 in Tandem position/4) Trunk rotation in Romberg position/5) Exercise 4 in Tandem position/6) Step forward over an obstacle/7) Step side over an obstacle/8) Side steps associated with knees flexion and extension/9) Side steps associated with rise to toes/10) Exercise 8 to the right followed by exercise 9 to the left			
Session 9	exercises for upper limbs and upper trunk	3	Physical therapist 3
1) Coordination exercise involving changing arm positions/2) Coordination involving a different sequence of movements/3) Exercise 2 with more challenging sequence/4) Coordination alternated hand positions/5) Trunk rotation associated with shoulder abduction/6) Shoulder horizontal abduction and adduction and shoulder flexion and extension/7) Trunk lateral flexion/8) Trunk rotation associated with shoulder circumduction/9) Exercise 8 associated with trunk anterior inclination/10) Exercise 8 associated with trunk extension and shoulder flexion			
Session 10	exercises for lower limbs and lower trunk	3	Physical therapist 3
1) Ankle circumduction/2) Ankle plantar flexion associates with shoulder flexion/3) Coordination exercise involving both lower extremity movements/4) Crossing legs associated with trunk rotation/5) Exercises 2, 3 and 4 associated/6) Sit to stand/7) Exercise 6 with hip flexion when standing/8) Hip flexion and abduction associated/9) Exercise 8 associating opposite upper extremity movements/10) Lunges			
Session 11	dual cognitive and motor tasks	3	Physical therapist 3
1) Rise to toes + subtractions by threes/2) Knee flexion associated with ankle plantar flexion + naming objects beginning with letter P/3) Hip flexion associated with opposite shoulder abduction + naming water animals/4) Step forward associated with elbow extension + naming bakery items/5) Step forward associated with shoulder flexion + naming occupations/6) Exercises 4 and 5 associated + naming objects in alphabetic order/7) Static gait associated with upper extremity coordination movements + singing/8) Static gait associated with side step + naming famous actors/9) One leg stance balance moving the opposite leg forward and backward + naming flying animals/10) One leg stance balance moving the opposite leg in circumduction + naming famous singers			
Session 12	balance and gait exercises	3	Physical therapist 3
1) Balance maintenance in one leg stance position in a stair followed by stair step/2) Previous exercise associated with small lunges/3) Hip flexion touching the knee to maintain one leg stance/4) Balance maintenance in one leg stance position oscillating the opposite leg forward and backward/5) Previous exercise oscillating the opposite leg in a circumduction movement/6) Dynamic gait moving in a square shape/7) Dynamic gait moving in an X shape/8) Gait initiation, 3 steps forward and stopping/9) Gait crossing legs front and back/10) Exercise 8 and 9 combined			
Session 13	exercises for upper limbs and upper trunk	4	Physical therapist 4
1) Trunk lateral reach/2) Trunk rotation/3) Exercise 2 with coordination movements associated/4) Trunk anterior reach/5) Exercise 4 increasing range of movement/6) Exercise 5 with straightening spine reaction/7) Exercise 5 and 6 combination/8) Exercise 7 with shoulder abduction and adduction associated/9) Exercise 7 with trunk rotation associated/10) Exercise 8 and 9 associated			
Session 14	exercises for lower limbs and lower trunk	4	Physical therapist 4
1) Tandem sit to stand/2) Step forward associated with shoulder flexion/3) Side step associated with shoulder abduction/4) Step backward associated with shoulder flexion/5) Exercises 2, 3, 4 associated/6) Step forward associated with one leg stance/7) Side step associated with one leg stance/8) Step backward associated with one leg stance/9) Exercise 6, 7, 8 associated/10) Step backward associated with trunk rotation			
Session 15	dual cognitive and motor tasks	4	Physical therapist 4
1) Trunk anterior inclination followed by trunk lateral inclination + naming fruits forward and colors side/2) Hip flexion passing a ball from one hand to the other + naming occupations/3) Trunk rotation associated with shoulder flexion followed by side step + naming office objects/4) Side step associated with hands claps + naming jungle animals/5) Upper extremity coordination sequence + naming school objects/6) Step forward, side and backward + naming the months of the year in the inverse order/7) Side gait with upper extremity coordination movements + singing/8) Step forward and backward associating hip flexion and upper extremity coordination movements + singing/9) Side gait followed by forward and backward steps + singing/10) Previous exercise with more challenge sequence			
Session 16	balance and gait exercises	4	Physical therapist 4
1) Balance maintenance in Romberg position on a foam/2) Small squats in Romberg position associated with shoulder flexion and extension modifying center of gravidity on a foam/3) Trunk rotation in Romberg position on a foam/4) Step out of the foam forward and return to the foam/5) Step out of the foam side and return to the foam/6) Exercise 4 and 5 combined/7) Step out of the foam and return with coordination sequence movements/8) Static gait associated with boxing movements/9) Steps forward and backward associated with turning/10) Steps side associated with turning			
Session 17	exercises for upper limbs and upper trunk	5	Physical therapist 5
1) Coordination exercise involving change arm positions/2) Coordination involving a different sequence of movements/3) Exercise 2 with more challenging sequence/4) Coordination associated previous exercises/5) Coordination exercise combined with parts of the body/6) Trunk anterior inclination associated with trunk rotation/7) Exercise 6 with upper extremity movement/8) Standing exercises involving upper extremity movements in a wall/9) Exercise 8 associated with trunk rotation/10) Exercise 8 involving greater range of movements.			
Session 18	exercises for lower limbs and lower trunk	5	Physical therapist 5
1) Sit to stand and trunk rotation/2) Exercise 1 in Tandem position/3) Squats associated with trunk rotation/4) Knee flexion followed by step forward and upper extremity movements/5) March in place followed by side step/6) Step forward and side step associated/7) Side step followed by a hip circumduction/8) Step backward associated with trunk rotation/9) Step forward followed by a 180° turning/10) Exercise 9 with upper extremity movement associated			
Session 19	dual cognitive and motor tasks	5	Physical therapist 5
1) Sit to stand associated with forward step over an obstacle + naming salt foods/2) Sit to stand associated with side step over an obstacle + naming sweet foods/3) Exercises 1 and 2 combined + counting from sevens/4) Sit to stand associated with dynamic gait stepping over an obstacle + counting back by sevens/5) Dynamic gait associated to stepping over an obstacle and turning + naming hygiene objects/6) Dynamic lateral gait stepping over obstacles + naming flowers/7) Exercise 6 with increased challenge + naming trees/8) Dynamic lateral gait associated to steps forward between obstacles + naming objects starting with letter S/9) Dynamic lateral gait associated to steps backward between obstacles + naming objects starting with letter F/10) Exercises 9 and 10 combined + naming countries			
Session 20	balance and gait exercises	5	Physical therapist 5
1) Balance maintenance in Tandem position on a foam/2) Small squats in Tandem position associated with shoulder flexion and extension modifying center of gravidity on a foam/3) Trunk rotation in Tandem position on a foam/4) Hip flexion touching the knee to maintain one leg stance on a foam/5) Balance maintenance in one leg stance oscillation the opposite leg forward, side and backward/6) Dynamic gait stepping over multiple obstacles forward/7) Dynamic gait stepping over multiple side obstacles/8) Waive obstacles/9) Dynamic gait involving stepping forward and backward stepping over obstacles/10) Dynamic gait associated with balance in one leg stance.			
